# A Consumer Neuroscience Study of Conscious and Subconscious Destination Preference

**DOI:** 10.1038/s41598-019-51567-1

**Published:** 2019-10-22

**Authors:** Thomas Zoëga Ramsøy, Noela Michael, Ian Michael

**Affiliations:** 1 Neurons Inc, Taastrup, Denmark; 2 Integrative Center for Applied Neuroscience, Copenhagen, Denmark; 3 College of Communication and Media Science, Zayed University, Dubai, United Arab Emirates; 4 College of Business, Zayed University, Dubai, United Arab Emirates

**Keywords:** Decision, Human behaviour

## Abstract

In studying consumer behaviors, the inclusion of neuroscience tools and methods is improving our understanding of preference formation and choice. But such responses are mostly related to the consumption of goods and services that meet an immediate need. Tourism represents a consumer behavior that is related to a more complex decision-making process, involving a stronger relationship with a future self, and choices typically being of a higher level of involvement and of a transformational type. The aim of this study was to test whether direct emotional and cognitive responses to travel destination would be indicative of subsequent stated destination preference. Participants were shown images and videos from multiple travel destinations while being monitored using eye-tracking and electroencephalography (EEG) brain monitoring. The EEG responses to each image and video were further calculated into neurometric scores of emotional (frontal asymmetry and arousal) and cognitive load metrics. Our results show that arousal and cognitive load were significantly related to subsequent stated travel preferences, accounting for about 20% of the variation in preference. Still, results also suggested that subconscious emotional and cognitive responses are not identical to subjective travel preference, suggesting that other mechanisms may be at play in forming conscious, stated preference. This study both supports the idea that destination preferences can be studied using consumer neuroscience and brings further insights into the mechanisms at stake during such choices.

## Introduction

In understanding human preference formation and decision-making, one recent successful approach has been to combine a neuroscientific approach with the study of real-life choices such as consumer behaviors. This approach has demonstrated the brain mechanisms underlying attentional, emotional and cognitive responses that drive consumer choices, going under headings such as “consumer neuroscience” and “neuromarketing”^1–6^.

Previous studies in consumer neuroscience have primarily focused on consumption behaviors that are related to more immediate rewards such as food choices, product purchase, and luxury goods. In doing so, these studies have been successful in providing insights into the mechanisms of these types of consumer behaviors, and even be able to predict such choices up to several seconds before they occur or are consciously felt^7–9^. Conversely, fewer studies have looked at choices that are more future-oriented, such as which career path to take or where to travel for holidays.

The purpose of this study is to employ the same approach as previously done in consumer neuroscience studies to these types of behaviors, to better understand whether immediate emotional and cognitive responses to future choice options are related to subsequent choices. Here, we focus on travel destination preference as a model to understand this type of non-direct consumer preference formation and choice. This area falls in a broader area of destination marketing, which recently has seen the first steps of including neuroscience tools and insights^10,11^. To better situate the current study, we have provided a Supplementary Section that goes through the background of destination marketing and how the study of emotional and cognitive responses have been conceptualized and studied, ranging from qualitative research methods to the recent inclusion of neuroscience methods (see Supplementary Materials).

At the core of prior research on destination preference formation lies both theoretical and empirical research suggesting that destination preference both has conscious and subconscious components, but that our understanding of the role of the subconscious is woefully lacking. Hence, the current study aims to capture the subconscious emotional responses to destination marketing stimuli through images and videos, to test whether such measures predict subsequent self-reported destination preference. In this study, our basic assumption was that variations in SDP would also manifest as rapid emotional responses to visual representations of destinations.

## Methodology

This study involves a multi-modal approach including self-reported destination preference, eye-tracking measures, and neuroimaging measures of emotional and cognitive responses. In the following we present the participant selection, choice of stimuli, measures, and analytical approaches.

Institutional approval for this study was obtained from the Zayed University (ZU14_086a_F). All participants filled out an informed consent form, and all recorded data were anonymized as part of the data acquisition. All experimental procedures were performed in accordance with relevant guidelines and regulations.

### Participants

To test the conscious and subconscious emotional and cognitive destination responses we recruited participants from a local convenience sample of participants who were possible candidates for travel due to vacation, studies, and/or work (N = 32, 15 women, age mean ± std = 20.3 ± 1.9) in the larger Copenhagen Region, Denmark. All participants provided informed consent following the declaration of Helsinki prior to enrolling in the study.

### Stimuli selection

The destination marketing stimuli used were images, names, and promotion videos from travel destinations. These destinations were Abu Dhabi, Dubai, Hong Kong, London, Madrid, New York, Paris, San Francisco, Singapore, and Sydney. We used three independent raters to identify images according to whether they were representative and creatively similar. The images and videos used were selected using the following criteria:

- The creative image and video should be representative of the destination based on the elements in the image (e.g., symbols, flags, status/icons etc.).
- If possible, the creative image should be representative of materials provided by each representative destination (e.g. their travel agency or other tourism entity).
- The creative images were compared on visual aspects such as color composition and visual complexity, using the NeuroVision tool (https://www.neuronsinc.com/neurovision-app).

### Apparatus and procedure

After signing an informed consent sheet, participants were fitted with eye-tracking glasses and a mobile brain monitor. They then underwent eye-tracking and neuroimaging calibration procedures. We used Tobii Glasses Pro 2 eye-tracking system and an ABM X-10 electroencephalography (EEG) brain monitor. The eye-tracking was recorded using the Tobii Glasses Controller software (www.tobii.com) and the EEG signals were recorded using the B-Alert Lab software (www.advancedbrainmonitoring.com) running in a Windows 10 environment (www.Microsoft.com). The following specifications apply for the EEG recordings: Nine sensor sites were used following the 10–20 system, including Fz, F3, F4, Cz, C3, C4, POz, P3, P4, fixed gain referenced to linked mastoids.

Eye-tracking calibration was done with the 1-point fixation proprietary Tobii solution. Eye-tracking data were used to ensure that participants were indeed paying attention to the images and videos presented on the screen, but not analyzed specifically for this project.

For the EEG recording, linked reference electrodes were located behind each ear on the mastoid bone. Impedances were ensured to be below 40 kΩ for all sites before recording commenced, following the recommended levels through the ABM system (http://tinyurl.com/y2s9uplz). The EEG data acquisition was sampled at 256 Hz with a high pass filter at 0.1 Hz and a fifth order, low pass filter at 100 Hz. The EEG data were transmitted wirelessly via Bluetooth to a nearby laptop computer which stored the psychophysiological data. We then used ABM’s proprietary acquisition software for artifact decontamination algorithms for eye blink, muscle movement, and environmental/electrical interference such as spikes and saturations.

EEG calibration was done using functional localizer tests, based on the ABM B-ALERT calibration process. The acquisition of benchmark data was used to create individualized EEG profiles required for calculating emotional arousal and cognitive load scores. The benchmarking session included three separate tasks: The Three-Choice Vigilance Task (3CVT), the Verbal Psycho-Vigilance Task (VPVT), and the Auditory Psycho-Vigilance Task (APVT). Data recorded from these tasks were then used to individualize the algorithms by adjusting the centroids and through this produce the metric scores of arousal and working memory load, as described in a previously published protocol^12^. This algorithm was saved as an individualized definition file, which was used as a regressor when calculating and normalizing metrics.

EEG data were calculated into selected different “neurometric” scores, including frontal asymmetry, emotional arousal, and working memory load, as described in more detail below. Here, each participant’s benchmark was used as a calibration file upon which EEG data were normalized to scores ranging from 0 (minimum) to 1 (maximum). Each emotional and cognitive scores were calculated with a 1-second temporal resolution. This procedure allowed us to reliably track emotional and cognitive responses over time. Additional scores for distraction and drowsiness were calculated but not included in the analyses.

**Figure 1:**
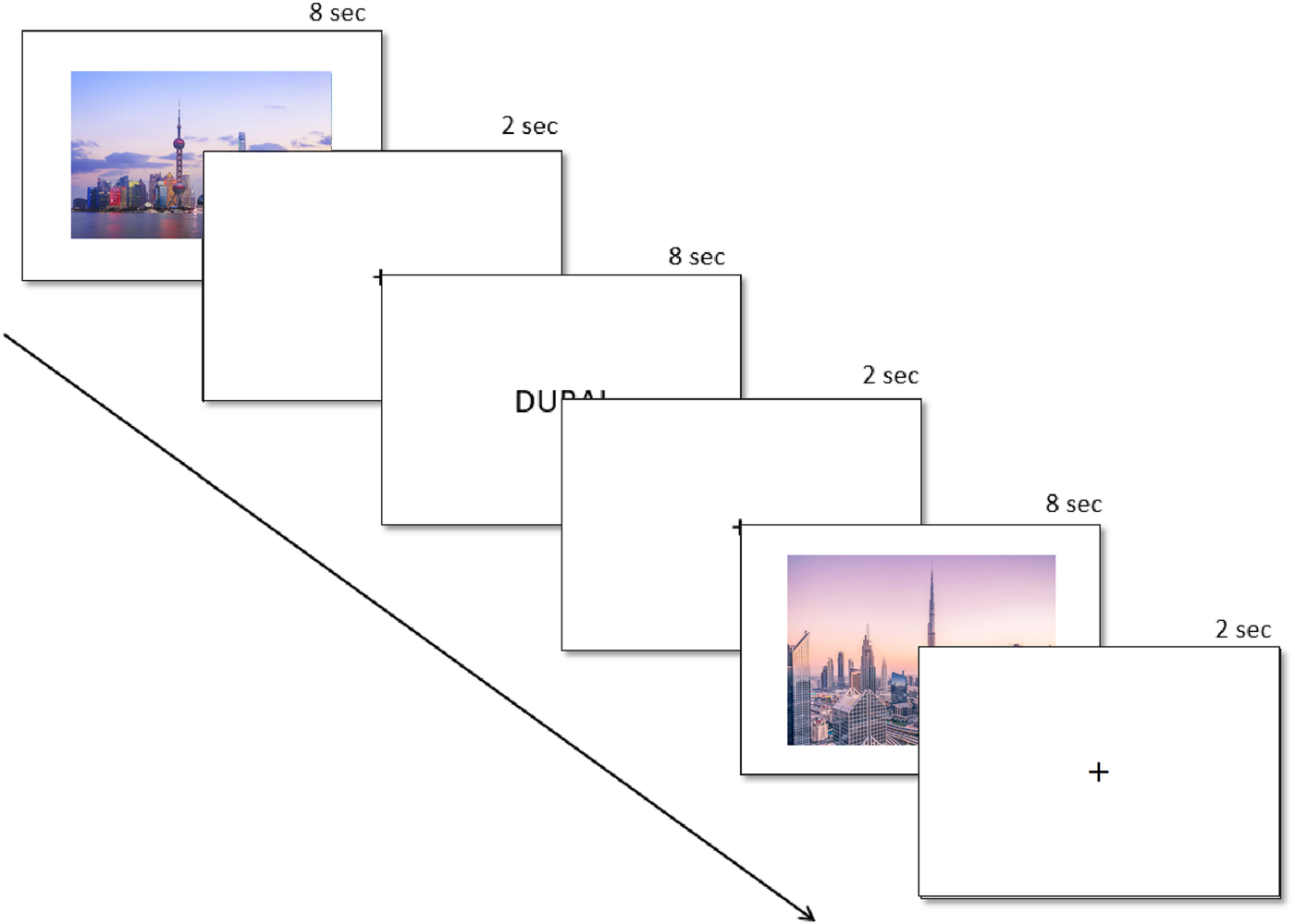
The study design, where images and names were presented for 8 seconds, and videos for the entirety of their duration (not shown). All stimuli were interspersed by an inter-stimulus interval of 2 seconds where a fixation cross was shown. Images in the photo are examples taken by Edward He and ZQ Lee on unsplash.com.

Each participant was then presented with several images, names and promotion videos from travel destinations. Images and destination names were presented for 8 seconds and videos for the duration of the video, separated by a 2 second inter-stimulus interval, while promotional videos were played in their full length (see Fig. 1). After the test, all participants underwent a surprise survey, which assessed their memory for destinations shown, conscious preference for traveling to the destination (“travel preference”) and destination associations. For the present study, responses to destination names are not included in the analyses.

All data were integrated, synchronized, and analyzed at the 1^st^ level using R v3.2.1 (www.R-Project.org) and a 2^nd^ level (group level) analysis was run in JMP v14.1 (www.jmp.com) running on a Windows 10 computer (www.Microsoft.com).

Emotional responses were calculated as frontal asymmetry and arousal scores based on previously published studies. Here, emotional valence and motivational direction was calculated based on the asymmetric engagement of the frontal part of the brain, as demonstrated by previous research^9,13–18^. The calculation used was based on prior studies using the gamma frequency band^8^, where the ratio between the mean power in the gamma band of frontal left electrodes (F3 and C3) relative to the mean of the right electrodes (F4 and C4), divided by the sum of both hemisphere pairs, and then normalizing the scores to a 0–1 range. On the normalized 0–1 range of scores, scores higher than 0.5 indicate increasingly positive scores and “approach motivation.” Conversely, scores lower than 0.5 denote increasingly negative emotional responses and “avoidance motivation.”

The second type of emotional response is referred to as emotional engagement or arousal, and reflects a bi-valent score that shows peak values for highly positive and highly negative events, and low scores for neutral emotions. The score was calculated as the posterior probability of arousal based on a neural network based model^12^ Arousal denotes emotional intensity but does not contain information about the actual direction of the emotional response^19–22^. Together, the arousal and frontal asymmetry scores provide a two-dimensional score for emotional responses. These two dimensions reflect neuroscience work showing that emotional responses can be evaluated on two dimensions: one dimension signifying the intensity of the emotion (here: “arousal”), and one denoting the positive-negative valence or direction (here: “frontal asymmetry”) of emotional responses.

The working memory load metric is a measure of mental processing load, i.e. the demand put on working memory, and increases when the amount of information being processed or kept active in memory is increased. The metric was calculated as the posterior probability of a given brain state to be in high workload, and thereby provide a continuous measure of working memory load^12^.

Finally, travel preferences were assessed through self-reported scores on willingness to travel to destinations, for vacation, studies, or work. Further analyses into the correlation between each of these scores were performed to assess whether they were highly correlated and would constitute a single type of destination preference, using both correlation analyses and Cronbach’s alpha.

## Results

Self-reported preferences showed a significant difference between destinations in terms of participants’ willingness to consider the destination for a vacation (F = 66.82, p < 0.0001), study abroad (F = 56.36, p < 0.0001), working abroad (F = 50.21, p < 0.0001) and recommending to others (F = 59.64, p < 0.0001). The responses to each destination were also highly correlated (Cronbach’s alpha = 0.85) suggesting that an aggregate score would be sufficient to capture self-reported measures of destination preference. To do this, we created an aggregate score of the four sub-scores (vacation, study, work, recommend) and named this the Travel Motivation Score (TMS). The TMS score was used throughout the rest of the study as a stated preference, to which we relate emotional and cognitive subconscious responses.

**Figure 2:**
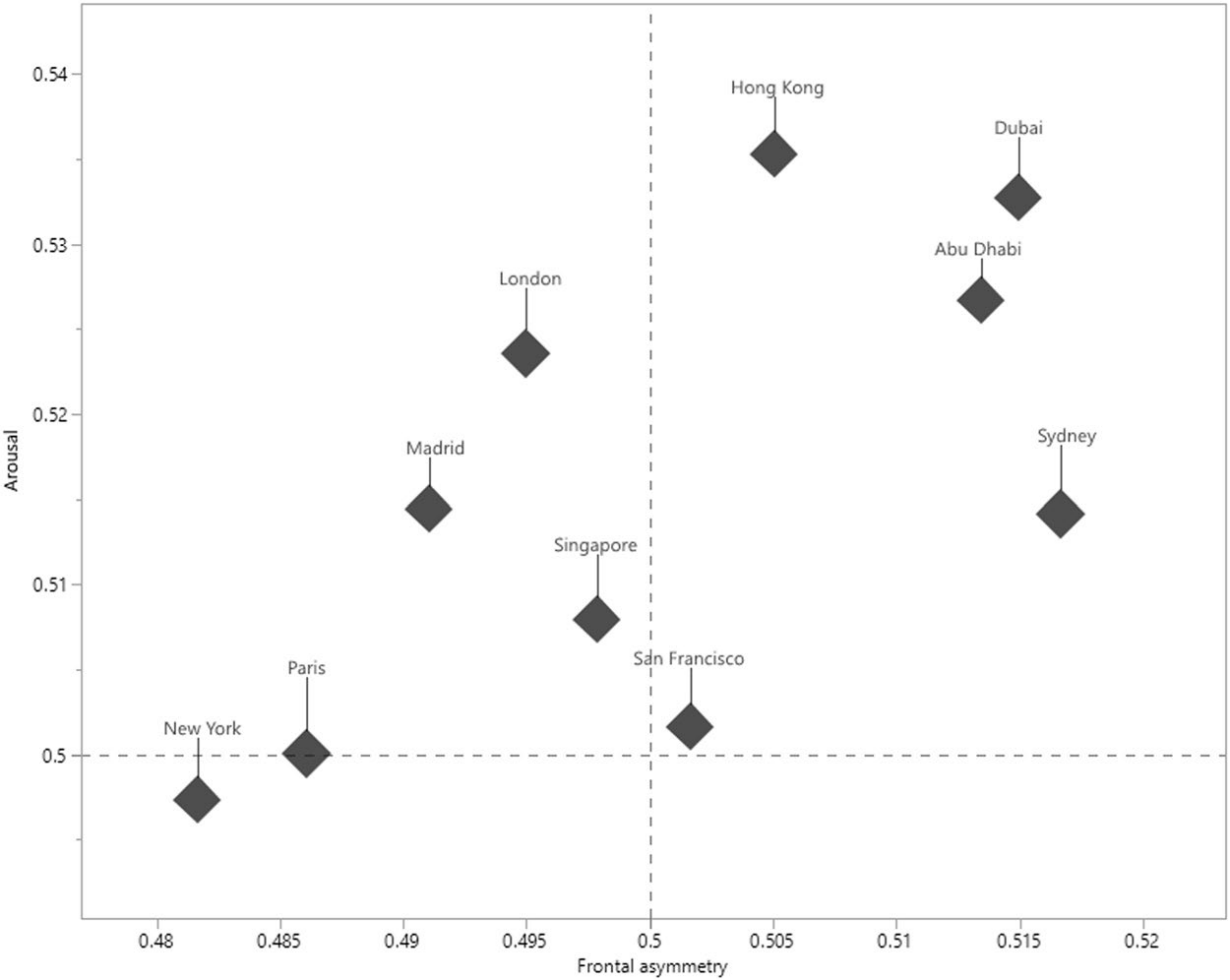
Distribution of emotional responses to travel destinations. The plot displays average scores for frontal asymmetry (x-axis) and arousal (y-axis) for each travel destination. Dotted lines are indicative of shifts between negative and positive emotions (x-axis) and low vs high arousal (y-axis). Destinations that score high on frontal asymmetry and arousal scores (e.g., Dubai) represent highly positive responses.

When looking at the emotional and cognitive responses we found a significant difference between the places on how they score, including frontal asymmetry (R^2^ = 0.029, F = 6.38, p < 0.0001), arousal (R^2^ = 0.009, F = 2.04, p = 0.0321), but not for cognitive load (R^2^ = 0.003, F = 0.80, p = 0.6142). Figure 2 shows the distribution of emotional responses to destinations.

**Figure 3:**
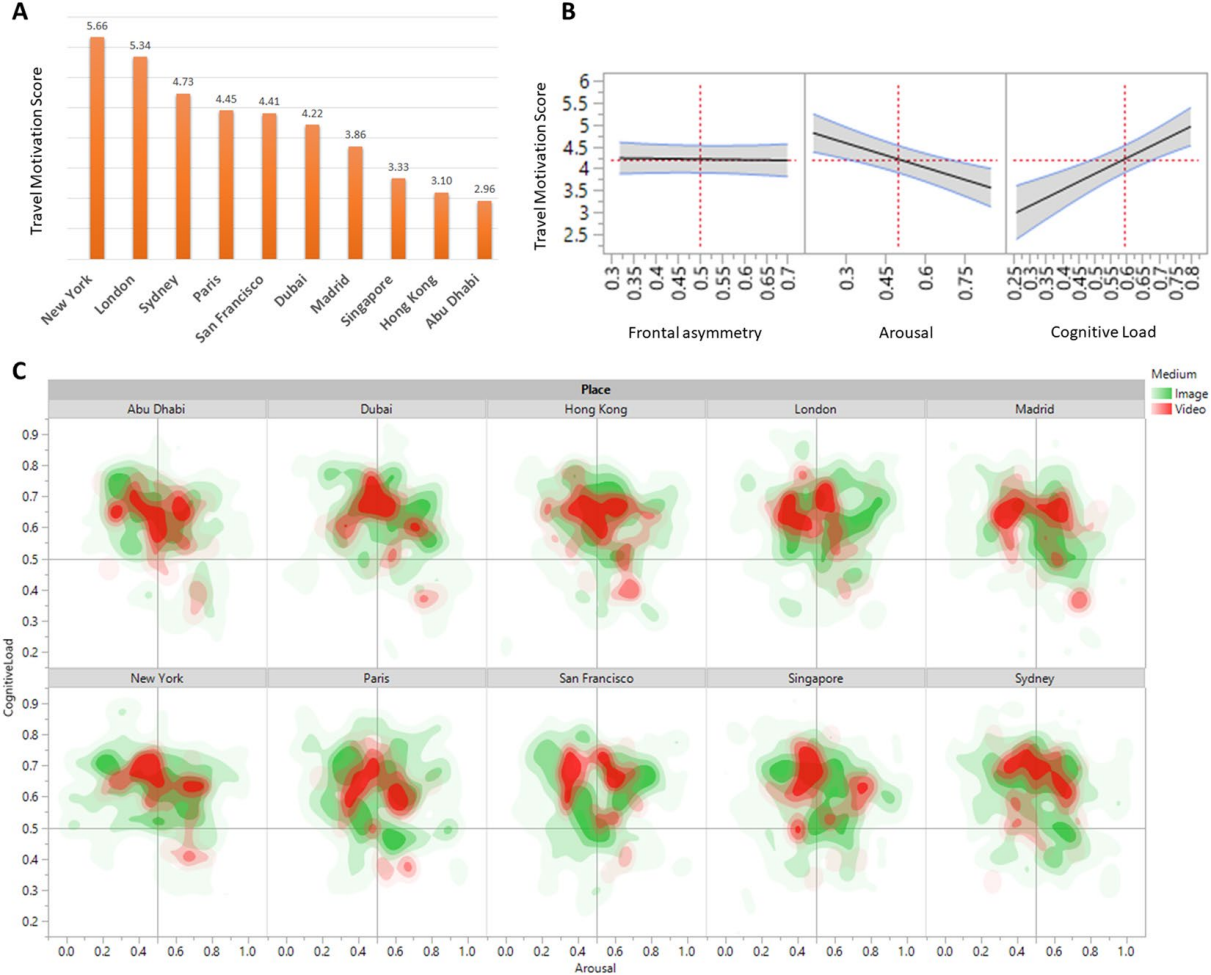
Distribution of emotional and preference scores between different destinations. (**A**) Distribution of average self reported travel preferences (TMS) for different destinations, showing that New York ranked highest and Abu Dhabi lowest on group averaged TMS. (**B**) Regression analysis results from the relationship between TMS and frontal asymmetry, arousal and cognitive load. Here, the black line represents the linear regression, gray area denotes the 95% confidence interval. (**C**) Contour plot shows the distribution of arousal (x-axis) and cognitive load (y-axis) scores for each of the travel destinations, using a Gaussian blur function and with intensity values going from low (light colors) to high (full colors), with further subdivision into responses for images (green) and videos (red). As this plot shows, image responses tend to be more variable than video responses.

We then tested whether emotional and cognitive responses when watching tourism images and videos were related to subsequent TMS scores. By running a random effects regression model we found that arousal (β = −1.858, F = 15.38, p < 0.0001) and cognitive load (β = 3.619, F = 21.06, p < 0.0001), but not frontal asymmetry (β = −0.136, F = 0.06, p = 0.8018), was related to subsequent TMS scores, and explaining almost 20% of the variation in TMS (model R^2^ = 0.193, RMSE = 0.46). Notably, arousal was negatively related to TMS and cognitive load was positively related to TMS. Figure 3 displays these effects along with the relative distribution of arousal and cognitive load scores for each destination.

A post-hoc exploratory analysis was then run to test for additional interaction effects. Here, we included frontal asymmetry, arousal, cognitive load and their interaction effects, and correcting for multiple comparisons using False Discovery Rate (FDR) correction. In doing so, arousal and cognitive load were still significant. In addition, a three-way interaction between frontal asymmetry, arousal and cognitive load (see Table 1). An exploration of the results showed a complex relationship between frontal asymmetry, arousal and cognitive load on predicting subsequent TMS. Motivation showed a positive relationship with TMS when arousal was low and cognitive load was high, and when arousal was high and cognitive load was low. Conversely, motivation showed a negative relationship with TMS when arousal and cognitive load were both either high or low.

**Table 1:** Results from the exploratory regression analysis, showing that besides the main effects of arousal and cognitive load, there is a significant three-way interaction between frontal asymmetry, arousal and cognitive load.

Term	Estimate	Std Error	DFDen	t Ratio	Prob > |t|
Intercept	3.651	0.65	505.0	5.59	<0.0001
Frontal asymmetry	−1.003	0.58	1900.0	−1.73	0.1357
Arousal	−1.813	0.48	881.3	−3.76	0.0004
Frontal asymmetry * Arousal	−6.794	4.09	1902.3	−1.66	0.1357
Cognitive Load	3.351	0.80	316.5	4.16	0.0003
Frontal asymmetry * Cognitive load	−1.312	4.83	1905.5	−0.27	0.7861
Arousal * Cognitive load	4.502	3.66	1588.2	1.23	0.2552
Frontal asymmetry * Arousal * Cognitive load	−113.770	29.84	1897.6	−3.81	0.0004

All p-values are reported after FDR correction for multiple comparisons.

Exploring the data further, we ran analyzes separately on images and videos. Here, we found that the emotional effect is only significant for videos (R^2^ = 0.139, F = 6.81, p = 0.0095) but not images (R^2^ = 0.173, F = 1.41, p = 0.236). These results indicate that differences in emotional responses to destinations are driven only by watching videos, suggesting that videos are more emotionally engaging than single images. There may be a number of ways to explain these differences: first, a single video collectively contains quantitatively more visual materials than single images do. Second, videos contain moving images which may be more visually engaging to look at. Third, videos include auditory elements such as voices, sounds and other elements that can produce and increase emotional responses.

## Conclusion

This paper contributes to the scientific literature in at least two ways. In one line of conclusions, it provides among the first insights into the basic mechanisms of the subconscious processes that underlie destination preference formation, and the distinction between subconscious and conscious processes. This paper suggests that there is a distinction between subconscious emotional responses and overt destination preference. Indeed, in the study of consumer psychology in conjunction with neuroscience, also known as consumer neuroscience, studies have repeatedly demonstrated a distinction between a subconscious “wanting” system and a conscious “liking” system, and that these systems contribute differently to consumer behavior and choice. The present study findings suggest that there may be different mechanisms at stake in driving emotional responses and overt preference ratings. As this study did not include any overt choice, an obvious next step in research is to conduct studies that include an element of choice, in which participants make actual overt destination choices. Here, based on both our results, and prior literature, we could contend that emotional responses during video/image perception may be significantly related to actuated choice, and that a conjunction between subconscious and conscious scores may be more predictive of actual choice than any scores individually. This is in line with prior consumer neuroscience studies on choice studies on choice^7,23–72^.

Another line of implications of this research is how it can influence the study of consumers’ minds. Understanding consumption behavior, from tangible choices of food to more intangible and future goods such as travel and insurance, requires testing of such choice. Here, our study contributes to the understanding of more abstract and future-oriented choice through the study of destination preference formation. While our study was not designed to include a final choice, the results are highly relevant to our understanding of preference formation in these conditions. The finding that customers display subconscious emotional responses that are not related to conscious destination preference confirms prior findings and ideas about a dual-system for decision-making.

While the present study demonstrates the feasibility of using neuroscience to inform destination preferences, a few limitations should be noted. First, this study only focused on general measures of emotional and cognitive responses, and did not include any level of spatial reconstruction of where in the brain the given activity was found. Subsequent studies should consider using neuroimaging methods that allow a higher spatial resolution and reconstruction, such as functional Magnetic Resonance Imaging (fMRI), high-resolution EEG (e.g., allowing for LORETA or other reconstruction methods), and magnetoencephalography (MEG). Such studies are expected to provide a better understanding of the neural mechanisms underlying destination preferences, and to what extent they overlap with other comparable consumer-related choices.

Another notable issue in the present study is that the stimulus materials diverged on the type and number of senses that were affected. Pictures are perceived visually, while videos contained music and narration in addition to the visual materials. While the present study was not aimed at testing for the effects of additional sensory information on emotional and cognitive responses and destination preference formation, future studies should seek to better understand how multimodal vs unimodal perception can affect destination preference and choice.

Finally, in the present study, we did not test for the effects of attention on destination preference. Since all stimuli were presented on-screen during a highly controlled setting, we would expect little variance in on-screen activity that was related to such preference. Also, for the present study, we did not have any prior hypotheses related to attention to certain elements. Should such hypotheses be suggested (e.g., that attention to faces is positively related to destination preference) such answers would be possible to targeted, even with the present data set.

Taken together, our findings are in line with the literature and now extend such findings to more complex decision-making. Future studies should seek to also include destination choices that vary in the temporal dimension (e.g., comparing choices of planned travel in a year vs those that are spontaneous and instant) to better understand how subconscious and conscious processes contribute to actual destination choices.

## Supplementary information

Literature review, conscious and subconscious destination preferences

## References

[1] Bell L (2018). Beyond self-report: A review of physiological and neuroscientific methods to investigate consumer behavior. Front. Psychol..

[2] Hsu M, Yoon C (2015). The neuroscience of consumer choice. Current Opinion in Behavioral Sciences.

[3] Karmarkar UR, Yoon C (2016). Consumer neuroscience: Advances in understanding consumer psychology. Current Opinion in Psychology.

[4] Plassmann H, Ramsøy TZ, Milosavljevic M (2012). Branding the brain: A critical review and outlook. J. Consum. Psychol..

[5] Shaw SD, Bagozzi RP (2018). The neuropsychology of consumer behavior and marketing. Consum. Psychol. Rev..

[6] Solnais C, Andreu-Perez J, Sánchez-Fernández J, Andréu-Abela J (2013). The contribution of neuroscience to consumer research: A conceptual framework and empirical review. Journal of Economic Psychology.

[7] Knutson B, Rick S, Wimmer GE, Prelec D, Loewenstein G (2007). Neural Predictors of Purchases. Neuron.

[8] Ramsøy, T. Z., Skov, M., Christensen, M. K. & Stahlhut, C. Frontal brain asymmetry and willingness to pay. *Front. Neurosci*. **12** (2018). 10.3389/fnins.2018.00138 PMC5890093 29662432

[9] Ravaja N, Somervuori O, Salminen M (2013). Predicting purchase decision: The role of hemispheric asymmetry over the frontal cortex. J. Neurosci. Psychol. Econ..

[10] Bastiaansen M (2018). My destination in your brain: A novel neuromarketing approach for evaluating the effectiveness of destination marketing. J. Destin. Mark. Manag..

[11] Kotsi F, Balakrishnan MS, Michael I, Ramsøy TZ (2018). Place branding: Aligning multiple stakeholder perception of visual and auditory communication elements. J. Destin. Mark. Manag..

[12] Stikic, M. *et al*. Modeling temporal sequences of cognitive state changes based on a combination of EEG-engagement, EEG-workload, and heart rate metrics. *Front. Neurosci*. **8** (2014). 10.3389/fnins.2014.00342 PMC4220677 25414629

[13] Ohme R, Reykowska D, Wiener D, Choromanska A (2009). Analysis of Neurophysiological Reactions to Advertising Stimuli by Means of EEG and Galvanic Skin Response Measures. J. Neurosci. Psychol. Econ..

[14] Berkman ET, Lieberman MD (2010). Approaching the bad and avoiding the good: lateral prefrontal cortical asymmetry distinguishes between action and valence. J. Cogn. Neurosci..

[15] Coan JA, Allen JJB (2003). Frontal EEG asymmetry and the behavioral activation and inhibition systems. Psychophysiology.

[16] Davidson RJ (2004). What does the prefrontal cortex ‘do’ in affect: Perspectives on frontal EEG asymmetry research. Biol. Psychol..

[17] Plassmann H, Venkatraman V, Huettel S, Yoon C (2015). Consumer Neuroscience: Applications, Challenges, and Possible Solutions. J. Mark. Res..

[18] Winkler I (2010). Frontal EEG asymmetry based classification of emotional valence using common spatial patterns. Worls Acad. Sci. Eng. Technol..

[19] Vecchiato G (2010). Changes in brain activity during the observation of TV commercials by using EEG, GSR and HR measurements. Brain Topogr..

[20] Carver CS (2006). Approach, Avoidance, and the Self-Regulation of Affect and Action. Motiv. Emot..

[21] Deitz, G. D. & Coleman, J. T. EEG-Based Measures versus Panel Ratings Predicting Social Media-Based Behavioral Response to Super Bowl Ads. Source *J. Advert. Res*. **56** (2016).

[22] Johnson Robin R., Popovic Djordje P., Olmstead Richard E., Stikic Maja, Levendowski Daniel J., Berka Chris (2011). Drowsiness/alertness algorithm development and validation using synchronized EEG and cognitive performance to individualize a generalized model. Biological Psychology.

[23] dos Santos ROJ, de Oliveira JHC (2015). Eye Tracking in Neuromarketing: A Research Agenda for Marketing Studies. Int. J. Psychol. Stud..

[24] Michael N, James R, Michael I (2018). Australia’s cognitive, affective and conative destination image: an Emirati tourist perspective. J. Islam. Mark..

[25] Ramkissoon H, Uysal MS (2011). The effects of perceived authenticity, information search behaviour, motivation and destination imagery on cultural behavioural intentions of tourists. Curr. Issues Tour..

[26] Gartner WC (1994). Image Formation Process. J. Travel Tour. Mark..

[27] Kock F, Josiassen A, Assaf AG (2016). Advancing destination image: The destination content model. Ann. Tour. Res..

[28] Li S, Walters G, Packer J, Scott N (2018). Using skin conductance and facial electromyography to measure emotional responses to tourism advertising. Curr. Issues Tour..

[29] Li Q, Huang Z(Joy), Christianson K (2016). Visual attention toward tourism photographs with text: An eye-tracking study. Tour. Manag..

[30] Wang Y, Sparks BA (2016). An Eye-Tracking Study of Tourism Photo Stimuli. J. Travel Res..

[31] Koelstra S (2012). DEAP: A Database for Emotion Analysis;Using Physiological Signals. IEEE Trans. Affect. Comput..

[32] Hubert M, Kenning P (2008). A current overview of consumer neuroscience. J. Consum. Behav..

[33] Karmarkar UR, Plassmann H (2019). Consumer Neuroscience: Past, Present, and Future. Organ. Res. Methods.

[34] Rustichini A (2005). Neuroeconomics: Present and future. Games Econ. Behav..

[35] Li S, Scott N, Walters G (2015). Current and potential methods for measuring emotion in tourism experiences: a review. Curr. Issues Tour..

[36] Matukin M, Ohme R, Boshoff C (2016). Toward a Better Understanding Of Advertising Stimuli Processing. J. Advert. Res..

[37] Hulme OJ, Skov M, Chadwick MJ, Siebner HR, Ramsøy TZ (2014). Sparse encoding of automatic visual association in hippocampal networks. Neuroimage.

[38] Berridge KC, Robinson TE, Aldridge JW (2010). Dissecting components of reward: ‘liking’, ‘wanting’, and learning. Curr Opin Pharmacol.

[39] Haber SN, Knutson B (2010). The reward circuit: Linking primate anatomy and human imaging. Neuropsychopharmacology.

[40] Chua HF, Gonzalez R, Taylor SF, Welsh RC, Liberzon I (2009). Decision-related loss: Regret and disappointment. Neuroimage.

[41] Che-Ha N, Nguyen B, Yahya WK, Melewar T, Chen YP (2016). Country branding emerging from citizens’ emotions and the perceptions of competitive advantage. J. Vacat. Mark..

[42] Gunn, C. A. *Vacationscape: designing tourist regions*. (Van Nostrand Reinhold, 1988).

[43] Mao IY, Zhang HQ (2014). Structural Relationships among Destination Preference, Satisfaction and Loyalty in Chinese Tourists to Australia. Int. J. Tour. Res..

[44] Echtner CM, Ritchie JRB (1993). The Measurement of Destination Image: An Empirical. Assessment. J. Travel Res..

[45] Pezenka I, Buchta C (2012). Measuring the resemblance between pictorial and verbal city image spaces. Int. J. Cult. Tour. Hosp. Res..

[46] Manhas PS, Manrai LA, Manrai AK (2016). Role of tourist destination development in building its brand image: A conceptual model. J. Econ. Financ. Adm. Sci..

[47] Lee J (2014). (Jiyeon). Visitors’ Emotional Responses to the Festival. Environment. J. Travel Tour. Mark..

[48] Yüksel A, Yüksel F (2007). Shopping risk perceptions: Effects on tourists’ emotions, satisfaction and expressed loyalty intentions. Tour. Manag..

[49] Bigné JE, Andreu L, Gnoth J (2005). The theme park experience: An analysis of pleasure, arousal and satisfaction. Tour. Manag..

[50] Hosany, S. & Gilbert, D. Dimensions of Tourists’ Emotional Experiences Towards Hedonic Holiday Destinations. *SSRN Electron. J.*, 10.2139/ssrn.1871768 (2009).

[51] Han H, Jeong C (2013). Multi-dimensions of patrons’ emotional experiences in upscale restaurants and their role in loyalty formation: Emotion scale improvement. Int. J. Hosp. Manag..

[52] Lin IY, Mattila AS (2010). Restaurant Servicescape, Service Encounter, and Perceived Congruency on Customers’ Emotions and Satisfaction. J. Hosp. Mark. Manag..

[53] Prayag G, Hosany S, Odeh K (2013). The role of tourists’ emotional experiences and satisfaction in understanding behavioral intentions. J. Destin. Mark. Manag..

[54] Faullant R, Matzler K, Mooradian TA (2011). Personality, basic emotions, and satisfaction: Primary emotions in the mountaineering experience. Tour. Manag..

[55] Sheen M, Kemp S, Rubin D (2001). Twins dispute memory ownership: A new false memory phenomenon. Mem. Cognit..

[56] Dijkstra W, Smit JH, Comijs HC (2001). Using Social Desirability Scales in Research among the Elderly. Qual. Quant..

[57] Nederhof AJ (1985). Methods of coping with social desirability bias: A review. Eur. J. Soc. Psychol..

[58] Ambler T, Ioannides A, Rose S (2000). Brands on the brain: Neuro-images of advertising. Bus. Strateg. Rev..

[59] Goodall, B. How tourists choose their holidays: An analytical framework. In *Marketing in the tourism industry* (eds Goodall, B. & Ashworth, G.) 1–17 (Routledge, 1990).

[60] Decrop A (2000). Personal Aspects of Vacationers’ Decision Making Processes: An Interpretivist Approach. J. Travel Tour. Mark..

[61] Lin C-H, Morais DB, Kerstetter DL, Hou J-S (2007). Examining the Role of Cognitive and Affective Image in Predicting Choice Across Natural, Developed, and Theme-Park Destinations. J. Travel Res..

[62] Chon K-S (1992). Self-image/destination image congruity. Ann. Tour. Res..

[63] Sirgy MJ, Su C (2000). Destination Image, Self-Congruity, and Travel Behavior: Toward an Integrative Model. J. Travel Res..

[64] Tapachai N, Waryszak R (2000). An Examination of the Role of Beneficial Image in Tourist Destination Selection. J. Travel Res..

[65] Woodside AG, Lysonski S (1989). A General Model Of Traveler Destination Choice. J. Travel Res..

[66] Eringa K, Zhou S (2015). A visual analysis of a cultural tourism destination. Res. Hosp. Manag..

[67] Yüksel A, Akgül O (2007). Tourism management. Tour. Manag..

[68] Pieters R, Wedel M (2004). Attention Capture and Transfer in Advertising: Brand, Pictorial, and Text-Size Effects. J. Mark..

[69] Pan B, Zhang L, Law R (2013). The Complex Matter of Online Hotel Choice. Cornell Hosp. Q..

[70] Connell J (2005). Toddlers, tourism and Tobermory: Destination marketing issues and television-induced tourism. Tour. Manag..

[71] Hanefors M, Mossberg L (2002). TV travel shows — A pre-taste of the destination. J. Vacat. Mark..

[72] Goossens C (2000). Tourism information and pleasure motivation. Ann. Tour. Res..

