# Supplementary material for: A Consumer Neuroscience Study of Conscious and Subconscious Destination Preference
Source: Sci Rep. 2019 Oct 22;9:15102. doi: 10.1038/s41598-019-51567-1 (PMC6805896; doi:10.1038/s41598-019-51567-1)
Supplement: Supplementary file 1 — Literature review, conscious and subconscious destination preferences [file 41598_2019_51567_MOESM1_ESM.docx]

SUPPLEMENTARY MATERIALS

**A CONSUMER NEUROSCIENCE STUDY OF CONSCIOUS AND SUBCONSCIOUS DESTINATION PREFERENCE**

**Thomas Zoëga Ramsøy**

**Noela Michael**

**Ian Michael**

**Destination marketing and neuroscience**

From the perspective of destination marketing studies, it is known that marketers of tourist destinations compete fiercely with the hope of gaining a competitive advantage. As part of this process, marketers strive to enhance their destination image by attempting to identify effective marketing and branding strategies. As such, over the last few decades the importance of destination images has been one of the most intensively researched area of tourism marketing ^12^⁠. However, the influence that destination image has on tourist behavior has yet to be fully understood and there is a critical need for further research in this area ^13^⁠.

In the tourism research area, destination image is a concept that is comprised of three main components: a cognitive component, which includes a person’s knowledge, beliefs, thoughts and awareness of a destination; an affective component, which includes a person’s feelings and emotions about a destination; and a connotative component, which includes a person’s future intention to travel to a particular destination ^14^⁠. Although researchers have examined all three components, research into what factors influence the affective component is gaining momentum ^10,15^⁠.

Most assessment methods used to investigate the effect of destination image on tourism behavior have relied on self-report measures (e.g., questionnaires and interviews) which rely a person’s conscious assessment of their own perceptions and feelings ^10^⁠. However, Li et al. ^16^⁠ suggest that an understanding of subconscious emotional responses to destination images using psychophysiological measures (e.g., electrodermal activity, facial muscle activity, heart rate response, eye movements, pupil dilation) might provide valuable insight into the autonomic emotional responses that happen when consumers are exposed to a destination stimulus. Within the tourism arena only a handful of studies ^10,17,18^⁠ have used psychophysiological methods to investigate effects of destination image through exogenous cues such as photographic images and photographic images paired with text. Other than Bastiaansen et al. ^10^⁠ who explored emotional responses to different destination images, none of these studies combined the use of neuroimaging methods, such as EEG, with eye-tracking methods, or used neuroimaging methods to investigate the influence of subconscious emotional responses to destination images on destination preference.

The purpose of this paper is twofold. First, it is intended to make a new empirical and theoretical contribution to our understanding of destination preference formation and choice. Second, it serves to demonstrate the additional value of using consumer neuroscience as an approach in understanding a broader palette of consumer behaviors, in particular tourism and destination choices. To achieve this, in this study we employ a combination of eye-tracking and EEG brain scanning to produce a paradigm which, to the best of the authors’ knowledge, has not been used previously in a tourism context.

Before presenting the results of the study, it is crucial to present a review of the most central themes and findings within the converging disciplines of tourism research and consumer neuroscience.

**Literature Review**

*Emotions*

Ubiquitous to all cultures, emotions are a complex stimulus response which include subjective feelings, physiological (body) responses, and expressive behavior (Ekman, 1999). When studying emotional responses, most consumer research studies have focused on facial expressions and speech patterns ^19^⁠. Over the last decade, however, an emerging literature combining economics, psychology, and neuroscience has emerged. Going variably and jointly under the headings of “neuromarketing”, “consumer neuroscience” ^3,4,20,21^⁠^3,20,21^, and “neuroeconomics” ^22^⁠, research in these areas has relied on two main sources for studying and understanding consumer choice. The first uses measures of consumers’ physiological responses to relevant behaviors and stimuli ^23–25^⁠, such as pupil dilation ^26,27^⁠, and heart-rate and respiration ^25^⁠. The second uses emotional responses in the brain to stimuli such as branding ^28^⁠ and attempts to predict consumer choice based on emotional responses ^9^⁠. Together, the results of research in these areas suggest that emotional reactions, measured by neural and physiological responses, can greatly help our understanding of consumer behavior.

Notably, in the neuroscience literature, there is a distinction between predicted and experienced outcome, as seen in the economic literature between predicted and experienced utility. Indeed, neuroscience research has suggested a distinction between two different motivational systems: a “wanting” system that is largely subconscious, and a “liking” system that is related to conscious, experienced utility ^4,29^⁠. These two systems are also found to be dependent on two separate neural networks: the “wanting” system relates primarily to the basal ganglia and amygdala functions ^4,30^⁠, and the “liking” system relates more to the ventromedial prefrontal cortex and insula ^31^⁠. Although what you “like” is often closely connected to what you “want”, there are instances that suggest independent and often conflicting motivations: for example, when you want to quit smoking (a “liking” motivation) but are driven by urges to smoke (a “wanting” motivation); or when you want to diet but feel urges for food that is high in calories. In this study, the intention is to apply a dual-process approach to measuring customer motivation, which combine self-reported preference (liking) with measures of emotional responses (wanting). In light of this discussion about a dual-process model of emotion and motivation and the overlapping yet distinct processes that explicit and implicit preference formation provides, it is important to note that implicit results from neuroscience measures have been shown to not only predict explicit self-report preferences but also to provide distinct response patterns connected to choice-related behaviors. This further enables us to predict emotional responses that travellers are likely to have *prior* to travel, and so provide an unparalleled insight into what drives travellers’ preference formations.

Taken together, there is a need for better understanding the emotional responses that consumers experience when exposed to the primary sensory inputs of visual and auditory stimuli (images, printed names, and videos) prior to visiting (or having visited) a travel destination and how these stimuli may emotionally trigger motivation to travel to that destination.

*Destination image and emotions*

In studying destination preference formation, emotions have been found to play an important role ^32^⁠. The image a potential tourist has prior to travelling to a destination can be either induced or organic ^33^⁠, which in turn influences their destination preference ^34^⁠. The induced image is often formed as a result of exposure to external sources such as travel brochures, pictures, photographs, videos, and guidebooks, while an organic image often develops internally from non-commercial sources, such as the opinions of family or friends, the media, and educational sources ^35^⁠. In this study, we focus on the impact of the induced destination image created during the *pre-visit* stage which influences the potential tourist's behavioral intentions towards choosing the travel destination.

Within the tourism context, the induced image evokes a first impression of a destination ^36^⁠. Moreover, given that the tourism experience is intangible, it would be worth understanding the role that the induced image plays in creating and conveying information ^17^⁠ and the emotional responses made towards those images ^11^⁠. Indeed, tourists who watched the film The Bridges of Madison County (set in rural Iowa) were more motivated to visit the destination and immerse themselves in the romantic fantasy of the film’s love story rather than to see the actual bridge. This suggests that tourist behavior is more likely to be motivated by emotional responses to a destination image rather than the actual physical characteristics of the destination themselves ^37^⁠.

Studies of the role of emotion in tourism have focused on emotions in a number of contexts, including festivals ^38^⁠, shopping ^39^⁠, theme parks ^40^⁠, holiday destinations ^41^⁠, restaurants ^42,43^⁠, heritage sites ^44^⁠, and adventure tourism ^45^⁠, amongst others. Most of these studies have relied on self-report measures and interviews ^17^⁠ which can result in the exclusion of valuable emotion-related information. In these situations, subjective feelings cannot be observed and, instead, the person experiencing the emotion must describe it to others, and each person's interpretation and description of a feeling may be slightly different. A reported emotional response therefore may be misrepresented by the participant, leading the researcher to draw inaccurate conclusions. The emotion may also be distorted or misremembered and therefore non-representative of the respondent’s actual emotional response at the time of interest ^46^⁠. Emotions are also likely to fluctuate, so accurately capturing moment-to-moment responses or emotional changes across an entire experience can be difficult. Furthermore, the specific elements of a destination image that led to an emotional response may not be easily revealed ^16^⁠. Lastly, the tendency to present oneself in a more favorable light can often lead to a social desirability bias ^47^⁠ which can seriously undermine the validity of findings collected using self-reported measures such as surveys and interviews ^48^⁠.

However, research in the field of neuroscience indicates that individuals can experience different emotions without being consciously aware of them ^49^⁠. The use of neuroscience methods may therefore offer more discrete and objective measures in gaining a deeper understanding of the effects destination images exert on emotional responses of consumers ^16^⁠.

*Destination Preference*

In addition to the above discussion of understanding the formation of a destination image, extensive research has been conducted to explore the influence of destination images on people’s destination preferences. For example, Goodall ^50^⁠ point out that destination images that meet an individual’s preference reinforce the individual’s choice of that destination. In addition, according to Mao and Zhang ^34^⁠, destination preference is an antecedent of destination choice and is formed as a consequence of the destination decision making process. Indeed, destination preference is defined as “an attitude resulting from an explicit comparison process by which one destination is chosen over the other” ^51^⁠ and has been investigated in terms of destination loyalty, destination satisfaction, and brand selection ^52^⁠.

Researchers have also examined the relationship between destination image and destination preference, highlighting that this relationship is mediated by a tourist’s self-image of the destination concerned ^53,54^⁠. Furthermore, Lin et al.^52^⁠ point out that existing literature suggests that destination images shape individuals’ preferences as well as their decision to visit certain destinations ^50,55,56^⁠. For example, Kotsi et al. ^11^⁠ found that tourists were more committed to travel to places that had images or pictures of a destination that reflected their everyday lives. Thus, it would be worth investigating what are these emotions behind destination brand images that weave a compelling preference in choosing a destination for a holiday.

Li, Scott, and Walters^23^⁠ suggest that the literature pertaining to the relationship between destination image and subconscious emotional responses has largely been ignored. This might be due to the lack of appropriate methodological approaches which could potentially be resolved by using appropriate neuroimaging techniques ^10^⁠. Therefore, in response to the shortage of objective data, the current research draws on the use of neuroimaging techniques to examine how pictorial images of destinations, presented visually to individuals, can capture subconscious emotional responses to a destination and to test whether these measures can predict subsequent self-reported destination preference.

*Destination image, emotions and neuroscience approaches*

Destination image content of travel destinations, besides attracting attention of the consumer, can evoke emotions that induce the desire to travel and influence both destination preference and destination choice ^57,58^⁠. A study by Yüksel and Akgül^58^⁠ explored the potential power of images depicted in postcards to evoke the desire to travel to a destination. Their findings revealed that postcard images induced positive emotions in consumers and created a desire to travel. This study, however, did not measure whether the number of images (i.e., pictures) depicted in the postcard affected the type or magnitude of the emotional response or motivation to travel to a destination, and whether single-image postcards have a stronger impact on imagery processing and emotional responses than multiple-image postcards.

Destination image visual content (e.g., advertisements, brochures, pictures, postcards, movies, videos) has been found to be good at capturing consumers’ attention ^59^⁠. Insights into the impact of visual content on attention have largely been outside the tourism field, particularly when using neuroscience and neurophysiology methods ^18^⁠. To provide a more accurate understanding of emotional responses to destination images, Table S1 highlights a number of studies that have adopted neuroimaging strategies in recent years and includes the research questions as well as the measures used and findings of each study. For example, Li, Huang, Christianson ^17^⁠ (see Table S1) used eye-tracking techniques to examine consumers’ visual attention toward tourism photographs that had text naturally embedded as part of the landscape and how they perceive the advertising effectiveness of the photographs. The findings indicated that texts within the landscapes of tourism photographs drew a majority of the viewers’ attention and they also spent more time viewing these images with text in a known language and single textual messages compared to text in an unknown language and multiple textual messages. Similarly, Pan, Zhang and Law ^60^⁠ used eye-tracking to determine what attracts user’s attention, as measured by points of fixation, to hotel webpages. They also found the subjects were more attentive to text when pictures were also present. Wang and Sparks ^18^⁠ (see Table S1) used eye-tracking and self-reported measures to analyze the appeal of tourism images by comparing visual attention towards tourism pictures between Chinese and Australian consumers. The findings indicated that the two ethnic groups displayed different visual processing behaviors. In another study by Eringa and Zhou ^57^⁠, visual information was used to evaluate the motivating factors that lead travelers with different cultural background in selecting a travel destination by focusing on the different stages of travel, i.e. decision-making process; during the visit and after the visit. They found that visual material can help frame the experience during all stages, and captures the attention of potential travelers.

Bastiaansen et al. ^10^⁠ (see Table S1) investigated emotional reactions using EEG-based neuromarketing experiments to establish brain event-related potentials (ERPs) elicited by destination marketing content (e.g., stimuli depicted in movies and photos). One group of participants was shown an excerpt from the movie *In Bruges* depicting destination marketing content such as tourist attractions around the city. The other group was shown a movie excerpt from *The Rum Diary* which contained no destination marketing content. Both groups were then shown images of Bruges, as well as images of an unrelated tourist destination, matched by type (the Japanese city of Kyoto). The researchers concluded that watching the movie *In Bruges* induced positive emotional responses in the ERP record and had a strong attention capture owing to the increase in emotional salience, and indicating that emotions are predictors of purchase behavior. The experiment also supports the notion that using EEG-based neuromarketing techniques to study the effectiveness of destination marketing effectiveness is viable. While only a handful of research studies have been conducted so far on visual attention and emotional responses in the tourism field, these studies suggest further investigations into consumers' viewing reactions toward visual materials used in tourism destination marketing and branding are needed. Chen and Phou’s (2013) study supports the brand relationship theory, indicating that tourists form emotional relationships with destinations and, therefore, intention to travel. Destination marketers should therefore aim to satisfy the tourists’ emotional needs such as satisfying the need for visiting a destination for relaxation and beauty (Ekinci, 2003).

Li et al. ^16^⁠ (see Table S1) compared self-report measures with psychophysiological measures. Participants were first shown destination promotional videos (DPVs). The researchers used digital skin conductance (SC) and facial electromyography (EMG) methods, as well as self-report measures, to record participants’ emotional responses. The research found that individuals’ self-reported emotional responses to destination advertisements were consistent with the participants’ emotional peaks identified from psychophysiological responses. However, the psychophysiological methods were far superior to the self-report measures as they were more sensitive discriminators and were able to capture significant differences in physiological responses between emotional advertisements and the non-emotional advertisement, which was not possible to establish with self-report measures. Moreover, the psychophysiological measures were able to distinguish non-emotional appeals from emotional appeals, as well as differentiating between the two emotional appeals. The psychophysiological measures better distinguished between the two fundamental dimensions of emotion (i.e. pleasure and arousal). Conversely, it is worth noting that the neuroscience methods adopted in this study were able to indicate the valence or direction of participants’ emotional responses.

Although destination marketing organizations promotional techniques, movies ^61,62^⁠ and picture postcards ^58^⁠ have been used to study the emotional influence they play in destination preference, no study to date has empirically tested emotional responses to destination images or videos, and their relationship to destination preferences. While these prior studies have focused on different types of destination stimuli, little has been done to have a more comprehensive measure of emotional and cognitive responses.

In the study presented here, we demonstrate the relationship between emotional and cognitive destination stimuli responses to later stated destination preferences. As such, this represents three types of emotional responses, two of which are subconscious and one conscious. Counting as subconscious emotional responses we look at two dimensions of responses: “arousal” denoting the intensity of the emotion; and “valence” or “motivation” denoting the direction of emotional responses. An additional relevant brain response includes the ability to keep information active in mind for a few seconds, often referred to working memory, where changes in this load is often referred to as “cognitive load.” Counting as a conscious, stated emotion (or “feeling”), self-reported liking scores were used to assess the conscious feelings towards destination stimuli.

In light of previous research, the following hypotheses were proposed with regard to the effect of destination image on destination preference based on subconscious emotional arousal:

- H1: A self-reported destination preference is significantly related to emotional and cognitive responses to visual representations of destinations. This hypothesis is further subdivided into three hypotheses:
  - H1a: Destination preference is significantly related to emotional valence
  - H1b: Destination preference is significantly related to emotional intensity
  - H1c: Destination preference is significantly related to working memory

**Table S1 Studies using neuroimaging methods to study emotions and destination image**

| **Year & Author** | **Target Origin & Sample** | **Problem addressed/Purpose of the study specific to emotions** | **Measures** | **Neuroimaging &physiology method** | **Major findings** |
| --- | --- | --- | --- | --- | --- |
| 2018 – Bastiaansen et al., | University students | To examine the effect of a popular movie with destination marketing content on emotional responses to tourist destination pictures by directly measuring ERP component changes from the brain.  The study only measured short-term changes in emotional responses to destination images, | Destination image and emotions  Emotional responses to features of place  Emotional reactions to marketing materials | EEG scanning | The findings confirm that emotions are known predictors of purchase behavior and EEG-based experiments (neuroimaging) are suitable in destination marketing.  Emotional responses to destination stimuli are stronger after seeing a related destination movie  The responses are so fast thus indicating that these responses are truly subconscious/  emotional and not rationally or cognitively processed  The results demonstrated that the emotionally ‘invigorating’ effect between the movie watched and the related destination pictures shown later lasts for at least 5–10min. |
| 2016  Li, Walters, Packer and Scott | 38 university students and staff from an Australian university | To examine the use of psychophysiological measures in tourism and usefulness of skin conductance (SC) and facial electromyography (EMG) methods to track emotional responses to destination advertisements.  The study also compared self-report measures with psychophysiological  Measures | Emotional reactions to tourism advertising | Skin conductance (SC) and facial electromyography (EMG) | The study confirmed that psychophysiological were able to capture moment-to-moment emotional responses  Psychophysiological methods are useful in measuring emotional responses to tourism advertising |
| 2016  Li, Huang and Christianson | Chinese native speakers who did not understand Arabic | The study aimed to find out whether:  Viewers understanding of text language affects visual attention and their perceived advertising effectiveness to tourism photographs  The number of textual messages in tourism photographs significantly affects viewers' visual attention to and the perceived advertising effectiveness of the photographs | Understanding of text language; the number of textual image; visual attention and perceived advertising effectiveness  Images chosen were all of landscapes in China or in various Middle Eastern nations with one or more texts naturally embedded within them (i.e., as signs, etc.). Texts were either in Chinese or in Arabic, respectively. | Eye-tracking  Questionnaire survey used to measure perceived advertising effectiveness | Images that had text naturally embedded within the landscapes of tourism photographs significantly drew the majority of participants' visual attention, irrespective of whether or not the text language was understood or other components in the photographs or people  Viewers spent more time viewing photographs with text in a known language compared to photos with text in an unknown language.  Higher perceived advertising effectiveness was reported toward the photographs that  included text in the known language  Viewers spent more time viewing photographs with a single textual message than those with multiple textual messages |
| 2016  Wang and Sparks | Australian and Chinese university students | The study aimed to find out whether:  Natural or built images and higher or lower arousal images attract a higher number of fixations  Natural or built images and higher or lower arousal images attract a longer duration fixation  Attention and scanning patterns differences between both groups | Built/natural environment  Higher/lower arousal | Eye-tracking  Self-report measures | Attention to the photographic images varied, for both natural or built environment and a low or high level of arousal.  No main effect for the natural/built environment was found in terms of fixation.  Natural and high-arousal images command greater attention.  High-arousal images received more fixation counts than low-arousal images  Australians and Chinese differed in terms of how attentive they were to tourism image stimuli and what elements of the images they attended to.  Attention to an image (higher fixation count or duration) is not correlated with liking of the image |

To the best of our knowledge, no study has specifically measured subconscious emotional and cognitive responses in relationship to pre-visitation except the study by Bastiaansen et al. ^10^⁠ which used a neuroscience approach, albeit with a particular focus on testing the effect of a framing effect during destination marketing and the relationship to affective destination image. In fact, Prayag et al. ^44^⁠ point out that the majority of research in this area focuses on tourists’ post-visit image perceptions and there is still a need for studies to investigate the relationship between tourists’ pre-travel images and emotional experiences. They also emphasize that empirical research in tourism investigating the direct relationship between emotions and destination image is scarce. Further, Goossens ^63^⁠ proposed that instructing respondents to use enactive imagery had the potential to “intensify the consumer’s emotional responses, appraisals, and behavioral intentions” (p.314). Also, in light of the consumer neuroscience literature suggesting two distinct motivational systems, we lack a deeper understanding of subconscious emotional responses to destination preference, and how this relates to (and possibly predicts) overt, self-reported destination preference, as well as whether such emotional responses show distinct response patterns.
